# The role of air pollution in epilepsy: a better understanding is needed

**DOI:** 10.1055/s-0045-1809663

**Published:** 2025-07-02

**Authors:** Prem Jareonsettasin, John S. Ji, Xiaowen Zhou, Ding Ding, Josemir W. Sander, on behalf of the SdHIELD (Social determinants of Health In EpiLeptic Disorders) Research Group

**Affiliations:** 1 University College London (UCL), Faculty of Brain Sciences, Queen Square Institute of Neurology, London, United Kingdom.; 2 Chalfont Centre for Epilepsy, Chalfont St Peter, United Kingdom.; 3 Tsinghua University, Vanke School of Public Health, Beijing, China.; 4 Fudan University, Huashan Hospital, Institute of Neurology, National Center for Neurological Disorders, National Clinical Research Center for Aging and Medicine, Shanghai, China.; 5 Epilepsy Foundation of the Netherlands (Stichting Epilepsie Instellingen Nederland, SEIN), Heemstede and Zwolle, The Netherlands.; 6 Sichuan University, West China Hospital, Neurology Department, Chengdu, China.

**Keywords:** Epilepsy, Air Pollution, Particulate Matter, Social Determinants of Health, Epidemiology

## Abstract

Social determinants of health, including neighborhood and built environment factors, play a crucial but underexplored role in epilepsy incidences. Among these, air pollution emerges as a potentially-preventable driver of epilepsy and adverse health outcomes. Evidence is accumulating on the effects of air pollution on the brain, especially in stroke and neurodegenerative disorders; however, the specific impact on epilepsy remains underresearched, potentially due to the complexities of studying this condition. The present narrative review addresses a critical knowledge gap by exploring: 1) the role of air pollution in epilepsy epidemiology; 2) the biological mechanisms of air pollution in the brain in the context of epilepsy; and 3) how air pollution affects the management of people living with epilepsy. We outline vital questions and actionable interventions regarding the role of air pollution in epilepsy.

## INTRODUCTION

Air pollution is a potentially-modifiable environmental risk factor. It is a potentially-preventable driver of epilepsy and a mediator of unequal health outcomes. Despite growing evidence linking air pollution to adverse brain outcomes, its specific roles in epilepsy remain poorly understood. This knowledge gap persists due to several roadblocks: the inherent complexity of epilepsy as a heterogeneous condition for diagnosis, the episodic nature of seizures, and limited interdisciplinary collaboration between environmental health and neurological research. Compounding this issue, the broader landscape of epilepsy is evolving. For instance, the incidence of conditions such as hippocampal sclerosis, historically associated with refractory focal epilepsy, has declined over recent decades, 1 2 potentially reflecting changes in environmental exposures or healthcare practices. At the same time, rising socioeconomic inequality 3 and climate change threaten to exacerbate epilepsy outcomes 4 and the neurological impacts of air pollution.

Compared to other brain disorders, such as stroke or neurodegenerative diseases, the role of air pollutants in epilepsy has received much less attention. Nevertheless, emerging evidence suggests that air pollution significantly influences known risk factors for epilepsy, including inflammation, oxidative stress, and neurodevelopmental disruption, as well as outcomes in people living with epilepsy. Unlike other conditions, a predisposition to abnormal brain excitability is a core characteristic of epilepsy; yet, the specific mechanisms through which pollutants may exacerbate this vulnerability remain poorly studied. For instance, pollutants carried by particulate matter (PM), including heavy metals, persistent organic pollutants, and airborne microplastics (MPs), may interact with neurochemical pathways to heighten seizure susceptibility or interfere with antiseizure medications. These aspects, though critical, are underexamined ( Table 1 ).

**Table 1:** What is air pollution?

Air pollution is a heterogeneous mixture of gaseous components and particulate matter (PM). A distinction should be made between ambient and indoor air pollution because the sources and constituents significantly differ. The primary gaseous air pollutants are nitrogen oxides (NOX), sulfur dioxide (SO2), carbon monoxide (CO), ozone (O3), carbonyl compounds, and organic solvents. Particulate matter is made up of solid and liquid particles and categorized by size: coarse particles (PM10, diameter < 10 μm), fine particles (PM2.5, diameter < 2.5 μm), and ultrafine particles (PM0.1, diameter < 0.1 μm). Each PM category includes components from smaller-sized PM categories. The composition and sources of PM are diverse. Anthropogenic sources include biomass solid-fuel burning, fossil fuel combustion, vehicle emissions, industrial processes and agricultural activities. Natural sources include wildfires, dust storms, volcanoes, ocean splashes and biological aerosols. 80% of ambient PM comprises organic carbon (OC), elemental carbon (EC), salt, mineral dust, nitrite (NO3-), sulphate (SO42-), and ammonium (NH4 + ). Where people live and how they spend their time governs their exposure to different compositions and concentrations of air pollutants. Additionally, airborne microplastics are recognized as significant pollutants. Microplastics (MPs) are plastic particles smaller than 5 mm, derived from the degradation of larger plastics, primarily through solar ultraviolet (UV) radiation. Microplastics include polyethylene (PE), polymethyl methacrylate (PMMA), and polyethylene terephthalate (PET). Particulate matter and microplastics also act as vectors for other chemical compounds, particularly heavy metals and persistent organic pollutants (POPs). Common POPs include polychlorinated biphenyls (PCBs), Di(2-ethylhexyl)phthalate (DEHP), polybrominated diphenyl ethers (PBDEs), perfluoroalkyl substances (PFAS), bisphenol A (BPA), polychlorinated dibenzo-p-dioxins (PCDDs), and p-benzoquinone (PBQ).

Epilepsy is often a symptom of underlying determinants of health, adding to the complexity. The roles of social determinants of health (SDHs) in the epidemiology, biology and management of epilepsy are poorly understood relative to genetic or molecular underpinnings. Thus, understanding how air pollution influences the epidemiology, biology, and management of epilepsy is critical for developing effective interventions and addressing growing disparities. In the present study, we attempt to synthesize the seemingly-disparate streams of evidence into a coherent framework. We outline actionable questions to address the role of air pollution in epilepsy.

## EPIDEMIOLOGY

**Figure 1:**
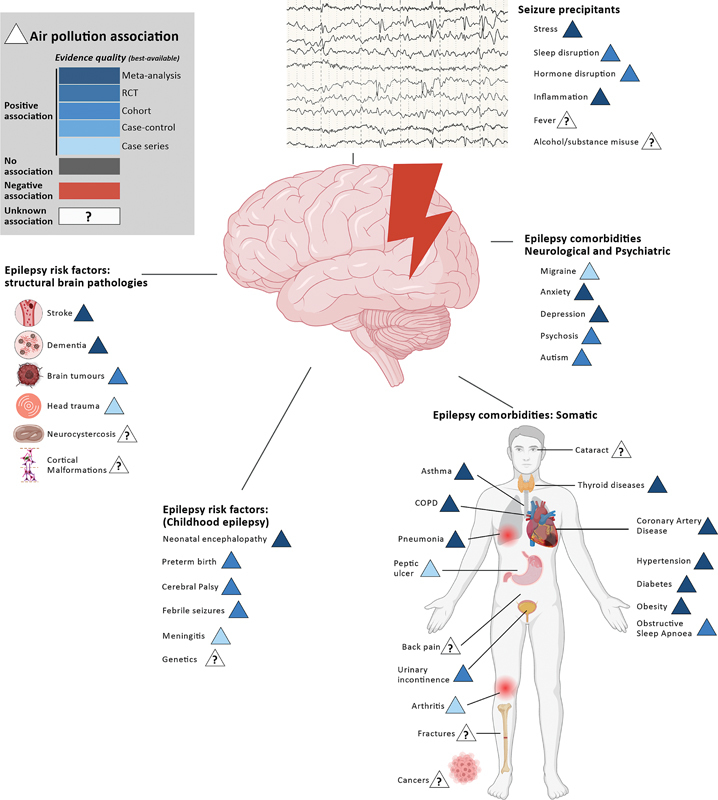
Notes: The circle indicates poverty; the triangle indicates air pollution; and different shades of blue indicate the quality of the best available evidence for positive associations. Epilepsy risk factors, comorbidities, and seizure precipitants associated with exposure to air pollutants.

Air pollution increases the risk of significant structural insults that predispose to epilepsy ( Figure 1 ). Short- and long-term exposure to ambient air pollutants (nitrogen dioxide [NO2], sulfur dioxide [SO2], ozone [O3], carbon oxide [CO], PM2.5, and PM10) increases the incidence of stroke. 5 The risk of brain tumors increases, dose-dependently, with exposure to specific pollutants. 6 Common sources of brain metastases—lung, breast, colon, thyroid and skin cancers—are increasingly linked to air pollutant exposure. This also applies to dementia, as one recent meta-analysis 7 shows the dose-related risk of dementia with PM2.5. The association with head trauma is not well assessed, with only one study 8 from Taiwan reporting that short-term exposure to PM2.5 and nitrogen oxides (NOx) is associated with an increased risk of traumatic intracranial hemorrhage. The mechanisms here are likely complex, with contribution of transient neuropsychological impairment that can increase the risk of head trauma.

The risk factors for childhood epilepsy are increasingly associated with exposure to air pollutants ( Figure 1 ). Long-term exposure to PM2.5 increases the risk of neonatal encephalopathy. 9 Exposure to PM2.5 and PM10 in the 1 ^st^ trimester, and to PM2.5, PM10, and O3 in the 3 ^rd^ trimester, increases the risk of preterm birth. 10 11 In a Canadian cohort study, 12 the average prenatal PM2.5 exposure increased the risk of cerebral palsy among full-term births A Danish cohort study 13 showed exposure to NO2 increases the risk of febrile seizures. Exposure to diesel exhaust particles increases susceptibility to invasive pneumococcal disease, a common cause of meningitis. 14 No study has explored the potential associations between air pollutants and cortical malformation, despite accumulating evidence of harmful effects on normal cortical development.

Hardly any investigations have explored air pollution exposure directly with epilepsy incidences and relative risks. One study 15 examined the association between the incidence and prevalence of epilepsy using Global Burden of Disease data with broad environmental indices of the surveyed countries. This study 15 suggested that the Air Quality Index was negatively associated with epilepsy prevalence but not incidence after adjusting for socioeconomic confounders. Interestingly, the association with the Pollution Emissions Index, which is typically measured at the source, was normal in this study. 15 This highlights the importance of investigating other environmental factors (such as weather conditions) that can concentrate pollution in certain areas. A single-center, case-control study 16 conducted in the Netherlands reported no association between new epilepsy diagnosis and the annual average O2, O3, PM2.5, and PMN10 exposure for the year of presentation to the clinic. Notably, the median levels of pollutants for cases and controls were low (such as PM2.5 of 14.2 μg/m ^3^ ).

Poverty could confound the relationship between air pollution and epilepsy by being independently associated with both variables. Studies 17 globally show that poorer neighborhoods are more likely to be exposed to higher pollutant levels. In low- and middle-income countries (LMICs), increasing air pollution parallels rising urbanization. Indoor air pollution is a significant concern, 18 particularly in low-income households that rely on biomass fuels such as wood, charcoal, and dry cow dung for cooking and heating. Living in poverty is also associated with exposure to harmful chemicals from various sources: pesticides used in agriculture, industrial pollutants, and chemicals from poorly-regulated waste disposal. Ever ubiquitous microplastics are ever more present in poorer regions. Prevalent and unique to LMICs are slums with extremely high levels of average PM2.5 that can go up to 110ug/m ^3^ (such as in Korogocho, Nairobi, Kenya). 19 Ventilation is often inadequate, and burning biomass solid fuel is nearly universal. Unable to move up the fuel ladder, households resort to unorthodox fuels, such as plastic, or employ ineffective interim solutions, such as forced-draft stoves.

## PATHOPHYSIOLOGY

### Animal models of air pollutant-related seizures

In a kainic acid (KA)-induced mice epilepsy model, 20 PM2.5 exposure (6 mg/kg, once every 3 days for 31 days) increased seizure frequency, duration and severity. Exposure to carbon black and zinc oxide nanoparticles increased seizure susceptibility and severity, as well as spontaneous recurrent seizures, in a KA- and pentylenetetrazole (PTZ)-induced mice model of epilepsy. 21 Acute (1 hour) O3 exposure reduced the latency to the first seizure and increased the spread of seizure in a rat amygdaloid kindling model. 22 However, 15-day (once-a-day) O3 exposure increased the latency to the first seizure in a PTZ-induced mouse model. 23 In a febrile seizure rat model, acute exposure to low concentrations of SO2 alleviated neuronal damage, while high concentration aggravated neuronal damage. 24 These seemingly contradictory results require further investigation. One possibility is that the brain can adapt to chronic or low dose exposure but is sensitive to acute fluctuations in or high levels of pollution.

Additionally, some persistent organic pollutants (POPs) and heavy metals can be proconvulsive. Bisphenol A (BPA) at doses above 250 ug/kg showed proconvulsant activity in penicillin-induced seizure models in male rats. 25 Prenatal exposure to polychlorinated biphenyl (PCB) increases the susceptibility to audiogenic seizures in adult rats. 25 Perfluoroalkyl substances (PFASs) provoke a seizurogenic effect in developing zebrafish larvae. 26 Prolonged cadmium exposure exacerbated seizure severity in a KA-induced seizure mouse model. 27 Exposure to PM2.5 increased hippocampal lead, aluminum, and manganese content in rats. 28 Prolonged exposure to lead resulted in dose-dependent increases in PTZ-induced seizure thresholds in rats and mice. 29 Arsenic decreased the latency to seizure onset and the time of death in a PTZ-induced seizure mouse model. 30 Methylmercury (MeHg) poisoning during development increased seizure susceptibility in various animal models. 31 Intracranial exposure to nickel and cobalt induced focal seizures in rodents, 32 and in, monkeys, cadmium, copper, nickel, antimony, mercury, and cobalt. 33

### Mechanisms that predispose to hyperexcitability

**Figure 2:**
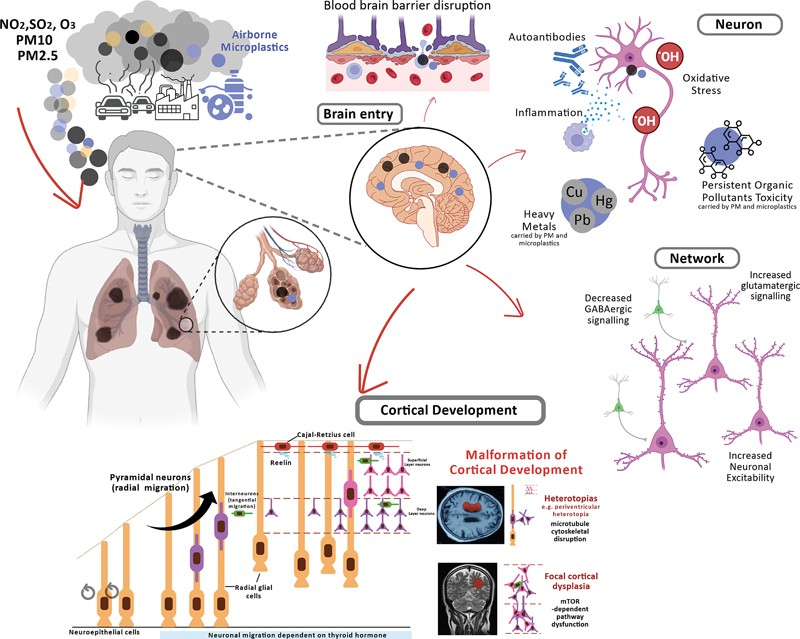
Air pollutants may predispose to seizures and epilepsy by disrupting normal neuronal function, network excitability, and cortical development. Air pollutants can affect the brain at all developmental stages. (Top Right) Acting directly in the brain, they can cause direct and indirect damage through oxidative stress, inflammation, mitochondrial dysfunction, altered myelination and alterations in normal cell signaling. Air pollutants indirectly affect the brain through systemic inflammation driven by chronic lung inflammation and damage to vital organs essential for healthy brain function. (Middle Right) Network excitability may be increased through multiple complex pathways that result in net excitatory-inhibitory imbalance, such as through modulation of glutamatergic, GABAergic transmission, ion channel, and neuronal excitability. Pollutants also indirectly modulate seizure threshold through autonomic disturbance, inflammation, sleep disturbance, and endocrine disruption. (Bottom) In the developing brain, disruption of normal neuronal migration pathways, increases in somatic mutation rates, maternal immune activation, placental impairment, and maternal thyroid dyshomeostasis are potential mechanisms through which air pollutants can cause permanent structural changes (that is, cortical malformations, MCDs).

Changes in neuronal excitability and excitatory-inhibitory neurotransmission balance contribute to seizure generation ( Figure 2 ). Air pollutants, especially CO, in people with epilepsy increased subclinical seizures on electroencephalograms (EEGs). 34 Human volunteers exposed to diesel exhaust for 30 minutes showed increased median power frequency in the frontal cortex. 35 Limited evidence 36 shows the potential of specific pollutants (such as SO2, some PCBs, polybrominated diphenyl ether [PBDE], and di(2-ethylhexyl)phthalate [DEHP]) to increase neuronal excitability ( Table 2 ). The effects are pollutant-specific, taking the different impacts of PCB19, PCB126, and PCB77 on human voltage-gated potassium channel 1.3 (Kv1.3). They are also concentration-specific (for example, MEHP inhibits calcium channels at lower doses and sodium and potassium channels at higher doses 37 ).

**Table 2:** Specific pollutant effects on neuronal excitability

Pollutant	Model system	Effect	Reference (see Supplementary Material*)
**Sulfur dioxide (SO2)**	Rat, hippocampal neuron	Shifts stead stage inactivation curve of transient outward potassium currents. Increases neuronal excitability.	1
	Rat, dorsal root ganglia	Inhibits voltage-gated sodium channel. Increases neuronal excitability.	2
**Polycyclic aromatic hydrocarbon (PAH):** **benzo[a]pyrene**	Mouse, hippocampal neuron	Represses synaptic vesicle exocytosis. Inhibits P/Q-type calcium channel.	3
**Polychlorinated biphenyl (PCB)**	Xenopus laevis oocyte	PCB19 inhibited human voltage-gated potassium channel 1.3 (Kv1.3) currents. PCB126 right-shifted steady-state activation curve of human Kv1.3. PCB77 enhances human Kv1.3 currents.	4–6
**Polybrominated diphenyl ether**	Rat, hippocampal neuron	PBDE 209 dose-dependent inhibition of voltage gated sodium channel. Decreases neuronal excitability.	7
**Bisphenol A (BPA)**	Mouse, dorsal root ganglia	BPA inhibits voltage-gated sodium channel.	8
**Di(2-ethylhexyl) phthalate (DEHP)**	Rat, hippocampal neuron	DEHP at 0.1 M and 0.3 M inhibit voltage-gated potassium channel of CA1 neurons. Mono-2-ethylhexyl phthalate (MEHP), the main active metabolite of DEHP, inhibits calcium channel at low dose, and sodium and potassium channels at higher dose of CA3 neurons.	9,10

Note: Supplementary Material containing further references available at: https://www.arquivosdeneuropsiquiatria.org/wp-content/uploads/2025/03/ANP-2024.0360-Supplementary-Material.docx .

The effects on excitatory glutamatergic and inhibitory gamma-aminobutyric acid (GABA)-ergic neurotransmitters are complex ( Table 3 ). The outcomes depend on the specific pollutant, exposure duration and protocol, model system tested, region of the brain, and sex of the animal. The source of pollutant matters: 24-hour exposure to PM2.5 collected from Los Angeles, 38 United States, increased the levels of N-methyl-D-aspartate receptors (NMDARs), whereas, from Shanxi, China, decreased NMDAR levels. 39 The duration of SO2 exposure above Environmental Protection Agency (EPA) standard levels also increases glutamatergic transmission after 1 week 40 but decreases it after 90 days. 41 Sex differences are essential: 6 days of exposure to PM2.5 increased hippocampal glutamate in males but decreased hippocampal GABA in females, for example. 42 Differential effects on different brain areas are not well assessed but exist:.10 days of exposure to polycyclic aromatic hydrocarbon (PAH) benzo(a)pyrene increased NMDA-R by 17 times in the hippocampus whilst decreasing by 4 to 35 times in frontal cortices. 42

**Table 3:** Specific pollutant effect on excitatory and inhibitory neurotransmission

Pollutant	Model system	Effect	Reference (see **Supplementary Material** *)
**Particulate matter (PM)2.5**	Mouse, hippocampal neurons	PM2.5 exposure (Shanxi, China) increases glutamatergic transmission via ROS-NF-kB.	11
	Macrophage and microglia cell cultures (human-derived)	PM2.5 exposure (Beijing, China) increases glutamatergic transmission via microglia-derived glutamate production.	12
	Rat, hippocampal neurons	24–48 hours of exposure to PM2.5 (Los Angeles, United States) increased N-methyl-D-aspartate receptor (NMDA-R) levels	13
	Rat, tracheal perfusion	12-week (1/week) exposure to PM2.5 (Shanxi, China) increases mGluR1 expression in male rats.	14
	Rat, Hippocampal slice	2 hours of exposure of PM2.5 (Los Angeles, United States) reduced synaptic function via nitrosylation of NMDA-R in CA1 but not dentate gyrus.	15
	Mouse, primary cortical neuron culture	24-hour exposure to PM2.5 (from Shanxi, China) decreased NMDA-R NR2B, PSD95 expression. PM2.5 collected in winter showed the strongest effect.	16
	Mouse, hippocampus	6-day (4 hours/day) exposure to PM2.5 (Boston, United States) to early postnatal (PND4-7,10-13) increased glutamate in the hippocampus of male subjects and caused loss of GABAergic neurons in the hippocampus of female subjects.	17
**Diesel exhaust particle**	Mouse, inhalation	8-week (5 hours/day, 5 days/week) exposure increases VGLUT1 levels in the prefrontal and temporal cortex. 2-month exposure (5 hours/day, 5 days/week) increases VGAT after 8 weeks in the prefrontal cortex and olfactory bulb, but not in the temporal cortex.	18
	Mouse, prenatal exposure	Prenatal 5-day exposure reduces NMDA-R expression in the hippocampus of male offspring.	19
	Cerebral organoid	Decreased relative GABA expression levels.	20
	Mouse, inhalation	2-month exposure (every 2 days) reduced GABA in the frontal cortex and hippocampus in a dose-dependent manner.	21
**Gasoline, vaporous**	Mouse	Acute exposure decreases GABA. Chronic exposure does not increase GABA.	22
**Sulfur dioxide (SO2)**	Rat, inhalation	1-week exposure to SO2 above Environmental Protection Agency (EPA) standards increases glutamate release, NDMA-R and PSD95 expression levels.	23
	Rat, inhalation	90-day exposure to SO2 above EPA standards decreases GluR1, GluR2, NR1, NR2A and NR2B expression in a dose-dependent manner.	24
**Polycyclic aromatic hydrocarbon (PAH):** **benzo[a]pyrene**	Rat	7-week exposure (daily) decreases glutamate levels, reduces GluR1 and GluR2	25
	Mice, intraperitoneal	10-day (1/day) exposure – NMDA-R1 expression increased by 17-fold in the hippocampus and decreased by 4-35-fold in the frontal cortex.	26
	Mice	3-day exposure increases NMDA-R.	27
	Mice, prenatal exposure	Prenatal exposure reduces NMDA-R NR2B subunit expression. Prenatal 3-day exposure downregulates AMPA-R expression.	28 29
	Mice, inhalation	Prenatal 3-day (4 hours/day, E14-E17) exposure increases prefrontal glutamate concentration.	30
	Rat, hippocampus	Increases gamma-aminobutyric acid (GABA) receptor expression.	31
**PAH** **1-nitropyrene**	Mouse, prenatal exposure	Reduced inhibitory synaptic transmission in the medial prefrontal cortex.	32
**Perfluoroalkyl substances (PFAS)**	Mouse, hippocampus	3-month (1/day) exposure Increased glutamate levels.	33
	Chicken embryo cerebellar granule neuron	PFOS exposure increases glutamate transmission.	34
	hiPSC	PFOS and PFOA inhibit human GABA-A receptor.	35
**Polybrominated diphenyl ether (PBDE)**	Mice	PBDE209 activates NMDA-R. PBDE47 prenatal exposure reduces AMPA-R.	36
	Xenopus laevis oocyte	6-OH-BDE-47 potentiates human GABA-A receptor.	37
**Di(2-ethylhexyl) phthalate (DEHP)**	Mouse, neuroblastoma cell line	Decreases synapsin1 and PSD95.	38
		Inhibits GABA-A currents.	39
**Polychlorinated biphenyl (PCB)**	Rat, X. Laevis oocyte	PCB138 and PCB180 prenatal exposure reduces glutamatergic transmission via NMDA-R. PCB52 increased GABA transmission.	40,41
	X. Laevis oocyte	PCB19, PCB28, PCB47, PCB51, PCB52, PCB95, and PCB100 potentiate human GABA-A receptors. PCB95 increases excitatory transmission.	37
**2,3,7,8-tetrachlorodibenzo-p-dioxin (TCDD)**	Rat	TCDD prenatal exposure reduces glutamatergic transmission via NMDA-R.	42
**Toluene**	Rat, hippocampal neurons	Acute toluene exposure increases the amplitude of NMDA-R currents and decreases GABA-R currents.	43
**Heavy metals**	Various	Manganese (Mn), lead (Pb), and mercury (Hg) can inhibit astrocyte glutamate transporter, increasing glutamate in the synaptic cleft.	44

Note: Supplementary Material containing further references available at: https://www.arquivosdeneuropsiquiatria.org/wp-content/uploads/2025/03/ANP-2024.0360-Supplementary-Material.docx .

The predisposition to seizures arises through mechanisms which increase the positive excitatory feedback loop. This can occur through the loss of inhibitory interneurons. Prenatal exposure to diesel, carbon black nanoparticles, MeHg, and 2,3,7,8-tetrachlorodibenzo-p-dioxin (TCDD) disrupts the normal development of fast-spiking parvalbumin (PV) interneurons. 43 Chronic inhalation of diesel particles increased the expression of perineuronal nets (found around PV interneurons) in adult mice's temporal cortex and transiently decreased PV interneuron numbers. 44 Another important mechanism is axon sprouting, which creates de novo recurrent excitatory circuits. Dose-related SO2 exposure resulted in mossy fiber sprouting in a rat febrile seizure model. 24 Other non-specific mechanisms associated with epileptogenesis and linked to air pollution include neuroinflammation, metal dyshomeostasis, disruption of normal blood-brain barrier, reactive astrocyte activation, and alterations in the gut microbiome. 45

### Impact on cortical development

Air pollutants disrupt normal cortical development, potentially causing cortical malformations such as heterotopias and focal cortical dysplasia ( Figure 2 ). Ultrafine particles, MPs associated with heavy metals, and POPs can cross the placental and immature blood-brain barriers. 45 Prenatal exposure to diesel exhaust disrupts normal *reelin* and heat shock pathways, which are critical for neuronal migration. 46 Volatile organic compounds, PAHs, PMs, and airborne MPs destabilized microtubule networks. 47 Heavy metals (such as cobalt, mercury, and cadmium) and POPs directly impair neuronal migration. 48 Lower thyroxine levels in first-trimester pregnant women are associated with air pollution exposure. 49 Transient modest maternal thyroid deficiency causes heterotopias. 50 Impaired placentation, the most common cause of chronic fetal hypoxia, can lead to polymicrogyria and heterotopia 51 Numerous meta-analyses 52 have linked air pollution with disorders of impaired placentation: preeclampsia, gestational hypertension, and intrauterine growth restriction. Air pollutants elevate maternal and fetal inflammation. A Mexican study, 53 particularly relevant to epilepsy, found higher titers of anti-glutamic acid decarboxylase 65 (GAD65) antibodies in children exposed to high PM2.5 levels in metropolitan areas.

## MANAGEMENT OF EPILEPSY

### Diagnosis and treatment

How air pollution affects time until diagnosis, misdiagnosis rates, and access to timely neurodiagnostic tests and specialist assessments are unknown. Air pollutants may affect the interpretation of neuroimaging findings. For example, quantitative hippocampal volume measures are commonly used in the assessment of temporal lobe epilepsy during the preoperative evaluation, but current assessment protocols do not currently consider the finding that PM2.5 levels are inversely associated with hippocampal volume, which may need to be taken into account when interpreting the significance of reduced volume. 54

Air pollutants are associated with common precipitants of seizures ( Figure 1 ): stress, sleep disturbance, inflammation, and endocrine disruption. Short-term PM exposure increases systemic inflammatory markers; 55 PM2.5, NO2, SO2 and O3 is associated with poor sleep quality; 56 and PM2.5, PAH and BPA decrease progesterone levels. 57 However, epidemiological evidence of increased seizure risk from pollutants is scarce and inconclusive. A small meta-analysis showed that short-term exposure to O3, NO2, and CO increased seizure risk, though the effect size was minimal for O3 and NO2. 58 A Danish cohort study 13 showed that exposure to NO2 was associated with a 5% higher risk of childhood febrile seizures.

What is more apparent is increased healthcare service use: PM10 is associated with increased emergency ambulance calls for epilepsy; 58 short-term exposure to NO2, SO2, CO, PM2.5, and PM10 increase hospitalization; 58 O3 is associated with reduced risk of hospitalization; 58 and NO2 and SO2 also increased visits to the outpatient epilepsy clinic in a single-center study. 59 The two studies 60 61 that explored seasonality showed the strongest associations in the winter months. Only two studies 62 63 have explored long-term exposure to pollutants and hospitalization. No studies have investigated the role of indoor household pollution.

### Outcomes

Air pollution may play a role in the effectiveness and toxicity of antiseizure medications (ASMs). Relevant to many common renally-excreted ASMs, (such as levetiracetam), short- and long-term air pollution exposures impair renal function. 64 Long-term exposure is associated with higher levels of liver enzymes and increased odds of metabolic dysfunction-associated fatty liver disease. 65 Particulate matter has also been shown to inhibit some *cytochrome P450s* 66 and induce others. 67 At least 3 PAHs induce *cytochrome P450 family 1* ( *CYP1* ) expression, which can be further augmented by valproate. 68 This is concerning because the most widely available first-line ASMs in LMICs (such as phenobarbital and carbamazepine) are not enzyme-neutral. Pollutants also interact directly with proteins associated with drug resistance, such as P-glycoprotein (P-gp) and multidrug resistant 1 (MDR1). Six hours of exposure to diesel exhaust particles increases P-gp levels, 53 while 24 hours of exposure decreases these levels. 69 Two weeks of exposure to ultrafine particles increased *ATP binding cassette subfamily B member 1* ( *ABCB1* [MDR1]) transporter levels in a mouse cortex. 70 Some pollutants, such as TCDD and PAH, can activate aryl hydrocarbon-receptor pathway, which increases MDR1 expression, 71 while others, such as microplastics and BPA, inhibit this pathway. 72

Seizure control alone does not guarantee a good outcome: air pollution is associated with an increased risk of developing multimorbidity 73 ( Figure 1 ). It increases the risk and severity of many neuropsychiatric comorbidities that impact quality of life and seizure control. Long-term exposure to PM2.5, PM10, NO2, O3 and CO increases the risk of migraine. 74 Short-term exposure to NO2 and SO2 is a potential precipitant of acute migraine attacks. 75 Several studies 76 have associated long-term air pollution with the risk of depression and anxiety. Short-term exposure to SO2 and NO2 increases the risk of hospitalization for depression and anxiety. 75 People with epilepsy have a higher suicide risk, and short-term exposure to NO2, SO2, and PM10 is associated with increased suicide risk, with the effect particularly pronounced among women and individuals with lower levels of schooling. 77 Neurodevelopmental outcomes, critical in children with epilepsy, are linked to prenatal exposure to air pollutants. These include intellectual ability, attention, and fine psychomotor development. Autistic spectrum disorder and attention deficit hyperactivity disorder (ADHD)-like behavior are also implicated. 78

Pollution-associated increases in respiratory and cardiovascular diseases are consequential. Untreated asthma increases the risk of epileptic seizures. 79 Chronic obstructive pulmonary disease (COPD) is an independent risk factor for stroke-related seizures. 80 Drugs commonly used to treat more severe respiratory diseases, such as montelukast, theophylline, and systemic corticosteroids, have potential drug interactions with common first-line ASMs. Air pollutant exposure increases arrhythmias and sudden cardiac death, and it has been hypothesized 81 that air pollution is an aggravating event in sudden unexpected death in epilepsy (SUDEP). Importantly, pneumonia and ischemic heart disease are the most common causes of premature death in people with epilepsy, even when seizure-free for decades. 82

## ADDRESSING THE ROLE OF AIR POLLUTION IN EPILEPSY

Emerging evidence suggests that air pollution may play a role as a risk factor in epilepsy. Within the symptom-component cause model of epilepsy, various factors act alone or in combination to contribute to the condition. We presented a body of evidence showing that air pollution is associated with increased rates and severity of many of these component causes, including the main risk factors for childhood and adult epilepsy. Preclinical studies demonstrate the potential of specific pollutants to contribute to generating a hyperexcitable brain that is predisposed to seizures and associated neuropsychiatric comorbidities. However, we need a far greater and more nuanced understanding of the role of air pollution in epilepsy.

Our current understanding is lacking in scope, specificity and scale:

- Scope: Key aspects and populations are missing from current epidemiological studies. Most studies do not report or control for indices of socioeconomic status (SES), a significant confounder. Temporal exposure characteristics are often unclear and masked by averages; we need to know peak and cumulative exposure at least. 83 Knowing when the risk of seizure is highest, such as hours or days after exposure, is crucial if we were to prevent seizures. The concentration-response associations are also unclear, and we do not know if the relationship is non-linear or if a threshold effect exists. Critical areas are missing from the field. First, how do pollutants influence epileptogenesis after the initial structural insult (such as acute stroke)? A potential window of opportunity to influence the outcome. Second, indoor pollution levels can be much higher, more toxic and more associated with SES. 18 What are the effects of indoor pollution? Third, what is the relationship between pollutants and drug resistance in epilepsy? Preclinical studies show potential interaction with many ASM molecular targets, pharmacokinetics, and drug-resistance proteins.
- Specificity: Air pollutant mixtures vary widely worldwide in their composition and concentrations due to different emission sources. A single pollutant and a mixture of pollutants are likely to affect outcomes differentially. This may also explain differences in biological responses when PM is taken from different locations and seasons in preclinical studies. Equally, epilepsy is heterogeneous. Focal epilepsy and generalized epilepsy have different biology, natural history, and responsiveness to ASMs. Preclinical studies hint at possible differences in pollutant effects on other brain areas. Pollutant effects on frontal lobe epilepsy versus temporal lobe epilepsy, or absence of seizures versus myoclonic seizures, could be very different. Precision medicine can benefit from more specific epidemiological studies.
- Scale: Scale is essential for understanding the full impact of air pollution on epilepsy risk and outcomes across diverse populations and regions. There are not many epidemiological studies on air pollution and epilepsies. The current studies are mostly small, single-centre, retrospective, and concentrated on a few countries, often near academic centers. The exposure timeframe is frequently too short, especially relative to the time course of the development of epilepsy (months to years). The range of pollution concentration is not broad enough. Two-thirds of people with epilepsy live in LMICs, where average PM2.5 concentrations are multitudes higher than in high-income countries (HICs). In contrast, the mean PM2.5 level of 49 ug/m ^3^ was calculated in a study 58 that included a recent meta-analysis reporting the 90 to 160 ug/m ^3^ range and conducted in 2023 in Dehli. 84 Premature and incorrect conclusions can be drawn if studies only focus on areas of relatively low pollution.

In conclusion, the role of air pollution in epilepsy represents an urgent and underexplored area of research, with a narrow window of opportunity to act. The broader trend of rising socioeconomic inequality 3 and climate change 4 is likely to adversely impact epilepsy outcomes and worsen the effects of air pollution. A more precise understanding at the epidemiological and mechanistic levels may enable us to mitigate and prevent the harmful effects. As seizures are a threshold phenomenon, even small interventions could lead to significant improvements.
